# Incomplete initial nutation diffusion imaging: An ultrafast, single‐scan approach for diffusion mapping

**DOI:** 10.1002/mrm.26894

**Published:** 2017-09-03

**Authors:** Andrada Ianuş, Noam Shemesh

**Affiliations:** ^1^ Champalimaud Neuroscience Programme, Champalimaud Centre for the Unknown Lisbon Portugal; ^2^ Centre for Medical Image Computing, Department of Computer Science University College London United Kingdom

**Keywords:** diffusion, magnetic resonance imaging, isotropic encoding, ultrafast MRI, mean diffusivity

## Abstract

**Purpose:**

Diffusion MRI is confounded by the need to acquire at least two images separated by a repetition time, thereby thwarting the detection of rapid dynamic microstructural changes. The issue is exacerbated when diffusivity variations are accompanied by rapid changes in T_2_. The purpose of the present study is to accelerate diffusion MRI acquisitions such that both reference and diffusion‐weighted images necessary for quantitative diffusivity mapping are acquired in a single‐shot experiment.

**Methods:**

A general methodology termed incomplete initial nutation diffusion imaging (INDI), capturing two diffusion contrasts in a single shot, is presented. This methodology creates a longitudinal magnetization reservoir that facilitates the successive acquisition of two images separated by only a few milliseconds. The theory behind INDI is presented, followed by proof‐of‐concept studies in water phantom, ex vivo, and in vivo experiments at 16.4 and 9.4 T.

**Results:**

Mean diffusivities extracted from INDI were comparable with diffusion tensor imaging and the two‐shot isotropic diffusion encoding in the water phantom. In ex vivo mouse brain tissues, as well as in the in vivo mouse brain, mean diffusivities extracted from conventional isotropic diffusion encoding and INDI were in excellent agreement. Simulations for signal‐to‐noise considerations identified the regimes in which INDI is most beneficial.

**Conclusions:**

The INDI method accelerates diffusion MRI acquisition to single‐shot mode, which can be of great importance for mapping dynamic microstructural properties in vivo without T_2_ bias. Magn Reson Med 79:2198–2204, 2018. © 2017 The Authors Magnetic Resonance in Medicine published by Wiley Periodicals, Inc. on behalf of International Society for Magnetic Resonance in Medicine. This is an open access article under the terms of the Creative Commons Attribution License, which permits use, distribution and reproduction in any medium, provided the original work is properly cited.

## INTRODUCTION

Methods enabling rapid acquisition of dynamic MRI data have greatly affected contemporary MRI. Functional MRI 1, 2, 3, 4, 5, hyperpolarized imaging 6, MR fingerprinting 7, and multidimensional NMR 8 are based on, and continuously benefit from, ultrafast acquisition schemes. In contrast, diffusion MRI (dMRI) methods 9, typically relying on single‐diffusion‐encoded schemes 10, are not usually acquired dynamically, but their ability to probe micro‐architectural features such as anisotropy 11, complex fiber configurations 12, 13, microscopic anisotropy 14, 15, 16, 17, and cellular‐scale dimensions 18, 19, 20 have made them widely applicable 21. A few examples include early stroke detection 22, 23, 24, white matter orientation mapping 25, studies of neuroplasticity 26, or detection of microstructural aberrations upon disease 27, 28.

Rapid and dynamic determination of diffusion‐derived metrics, however, is particularly important for diffusion functional MRI (dfMRI) [29, 30], a method aiming to detect neural activity through non‐blood‐oxygenation‐level‐dependent (BOLD) mechanisms. Diffusion functional MRI evidenced faster activation dynamics and more localized activation foci compared with BOLD, suggesting it may be more closely correlated with underlying neural activity 29, 30, 31. However, dfMRI's temporal resolution can be limited by the necessity to acquire at least two signals (one baseline image and one diffusion‐weighted image) for quantifying the apparent diffusion coefficient. To avoid excessive T_1_ weighting and severe degradation in image quality, dfMRI measurements are typically separated by at least repetition times (TRs) greater than 2 to 3 T_1_, imposing a practical limit on temporal resolution. Additionally, T_2_ variations can occur on the timescale of a typical TR, potentially biasing the measurement and complicating the interpretation of dfMRI 32, 33.

Isotropic diffusion encoding (IDE) based dMRI has recently re‐emerged as a valuable tool for speeding up the acquisition of a valuable rotationally invariant parameter of the full diffusion tensor: its mean diffusivity (MD). Isotropic‐encoding schemes have been proposed, for example, by Mori and van Zijl, who suggested the application of consecutive gradients along the laboratory x‐, y‐, and z‐ gradients 34. Topgaard used a similar diffusion encoding in a triple stimulated echo sequence 35. De Graaf et al extended this idea to MR spectroscopy 36, and gradient waveforms were optimized to improve IDE's efficiency 37. Other methods imparting different b‐values within a single scan (which, however, requires averaging for phase cycling) by making different coherence pathways sensitive to different b‐values have also been presented 38. More recently, Eriksson et al presented magic angle spinning of the q‐vector, an elegant IDE framework harnessing harmonically modulated gradient waveforms 39 or numerically optimized waveforms 40.

Here, we present a method called incomplete initial nutation diffusion imaging (INDI), which is designed to obtain both a baseline and a diffusion‐weighted image in a single shot without loss of signal‐to‐noise ratio (SNR) or temporal resolution. Nutation angles are tailored to keep a “fresh” longitudinal magnetization reservoir, such that it can be used for consecutive measurements separated by only a few milliseconds, mitigating potential biases in MD quantification as a result of time‐varying T_2_. The accuracy of INDI is validated in phantoms and in vivo on a preclinical system. Simulations analyzing INDI SNR considerations and future applications, especially via dfMRI, are discussed.

## METHODS

**Figure 1:**
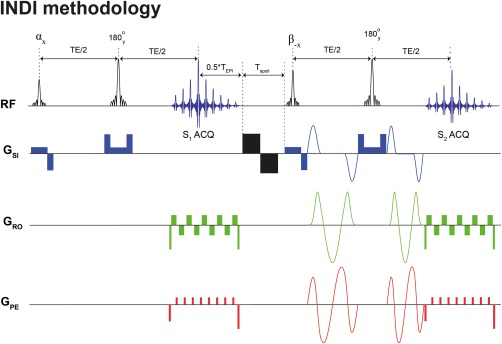
General INDI methodology. In this implementation, the sequence is furnished with isotropic diffusion‐encoding gradients. The sequence begins with an incomplete initial nutation (in our case, $\alpha_{x}=\frac{\pi }{4}$). A spin echo then proceeds, with the first acquisition providing the b = 0 s/mm^2^ image (in our implementation, an EPI acquisition, S_1_). Bipolar spoiler gradients (shown here in black) are then applied to remove residual magnetization, while refocusing the residual phase to remove possible nuisance artifacts, in a similar manner to phase rewinding in typical ultrafast imaging. Here, T_spoil_ was on the order of approximately 1–10 ms. The second nutation pulse (here, $\beta_{-x}=\frac{\pi }{2}$) rotates the remaining fresh longitudinal magnetization for the next spin echo, which is acquired with exactly the same timing and parameters as the first echo, only the diffusion gradients are now applied (S_2_). Here, we focus on obtaining the MD by applying IDE gradient waveforms. The resulting UF‐IDE pulse sequence thus provides both baseline and diffusion‐weighted images within $2TE+T_{EPI}+T_{spoil}$.

The INDI method is presented in Figure 1, and its theory is presented in the Supporting Information. The INDI mode of operation is rather simple: A fraction of the magnetization is rotated from the equilibrium position using a nutation angle *α*, leaving (ideally) an equal magnetization pool unperturbed; a non‐diffusion‐weighted image is then acquired. Immediately after this first acquisition, residual transverse magnetization is crushed, and all of the unperturbed magnetization in the “reservoir” is converted to transverse magnetization using a pulse angle *β*. An otherwise identical image to the previous excitation is acquired, but now the diffusion‐weighting gradients are also applied (Fig. 1). Thus, the two images required for quantifying diffusion coefficients are acquired with a separation of only a few milliseconds dictated by the TE, the EPI acquisition time, and the spoiler duration (see Fig. 1).

All experiments involving animals were pre‐approved by the institutional ethics committee. The phantom and ex vivo experiments were performed on a Bruker Aeon Ascend 16.4T scanner (Bruker, Karlsruhe, Germany) interfaced with an Avance IIIHD console and equipped with gradients capable of producing up to 3000 mT/m in all directions. In vivo experiments were performed on a Bruker BioSpec 9.4T scanner equipped with gradients capable of delivering up to 660 mT/m in all directions.

### Specimen Preparation

Doped water phantoms were prepared by gradually adding copper sulfate to a 30/70% (volumetric) D_2_O/water, until a longitudinal relaxation time of approximately 200 ms was obtained. The solution was placed in a 5‐mm NMR tube, which was sealed and placed in a 10‐mm NMR tube filled with Fluorinert (Sigma Aldrich, Lisbon, Portugal). Brain samples (n = 3) were extracted from healthy male C57bl mice weighing approximately 25 g by standard intracardial paraformaldehyde perfusion, followed by 12 h in a 4% paraformaldehyde solution at 4 ºC, and placement in phosphate‐buffered saline at 4 ºC. The brains were then soaked in a solution of phosphate‐buffered saline and 0.5M gadoterate meglumine (Dotarem, Guerbet, Lisbon, Portugal) at a dilution of 1:200 (2.5 mM) for 12 h 41, washed with phosphate‐buffered saline, and placed in a 10‐mm NMR tube filled with Fluorinert. All samples were allowed to equilibrate with the surrounding constant temperature of 37 ºC.

### Scout INDI Images

An INDI “scout” sequence was acquired once per specimen with all identical diffusion gradients turned off. These scouts were used to correct INDI‐derived maps.

### Water Phantom Experiments

Following the acquisition of routine localization images and shimming, the water phantom was subject to three types of experiments: a “ground‐truth” DTI, a conventional IDE MRI experiment, and the ultrafast IDE (UF‐IDE) sequence. All experiments shared the following acquisition parameters: single‐shot echo‐planar imaging (EPI), bandwidth = 652173 Hz, field of view = 10 × 10 mm^2^, matrix size 80 × 80 (partial Fourier encoding of 1.33, double sampled), leading to an in‐plane resolution of 125 × 125 µm^2^, with a slice thickness of 900 µm. The TR/echo time (TE) was 1800/20 ms. The UF‐IDE and IDE diffusion gradient waveforms were generated according to 42 for isotropic encoding. Diffusion gradients 40 placed before the INDI's second refocusing pulse (Fig. 1) were nulled to minimize TE, which can be done since the waveforms are independent and self‐refocusing the diffusion waveform following INDI's second refocusing pulse and had a duration of 7.5 ms and a b‐value of 400 s/mm^2^. The DTI experiments were performed using a pulsed‐gradient spin‐echo sequence with Δ/δ = 4/2 ms, and six diffusion‐weighted images (b = 400 s/mm^2^, gradients applied in non‐collinear directions) and six additional baseline images (b = 0 s/mm^2^) were acquired.

### Ex Vivo Brain Experiments

In the brain samples, IDE and UF‐IDE experiments were performed with identical acquisition parameters as described previously for the water phantom, but with the following modifications: slice thickness = 650 µm (six slices), b = 1000 s/mm^2^, and TR = 2500 ms; no double‐sampling was used.

### In Vivo Experiment

A male C57bl mouse weighing approximately 25 g was anesthetized with isoflurane (4% induction, 1–2% maintenance in 95% O_2_) and placed in the scanner. A closed‐loop circulating water system was used for temperature regulation, and respiration and rectal temperature were monitored continuously. Transmission was achieved through an 86‐mm quadrature resonator, and the signal was detected by a four‐element array receive‐only cryocooled coil (Bruker, Fallanden, Switzerland). The UF‐IDE and IDE experiments were performed using the following common parameters: fat‐suppressed single‐shot EPI, bandwidth = 326087 Hz, field of view = 16 × 12 mm^2^, matrix size 106 × 80 (partial Fourier encoding of 1.25), leading to an in‐plane resolution of 150 × 150 µm^2^; five slices were acquired, each 900‐µm thick, and one single field‐of‐view saturation slice suppressing signals from the head's ventral part was applied. The TR/TE for UF‐IDE and IDE were 1500/35 and 750/35 ms, respectively. Thirty‐two dummy scans were applied to reach a stable magnetization steady state. A b‐value of 1000 s/mm^2^ was achieved using an IDE waveform duration of 13.6 and 5.7 ms before and after the refocusing pulse, respectively, with a gradient peak amplitude of 610 mT/m. Another identical UF‐IDE experiment with 400 repetitions was acquired to assess the potential benefits of a recently developed denoising scheme 43.

### Analysis

Analysis in this study was performed using home‐written code in MATLAB (The MathWorks, Natick, MA). All images were analyzed with the raw data, without any further postprocessing. The full diffusion tensor was obtained from nonlinear fitting of the DTI data, and MD was calculated from the average of the eigenvalues. The IDE and UF‐IDE experiments provided the MD directly from $MD_{IDE}=-\frac{1}{b}\text{log}(S\left(b\right)/S\left(b=0\right)$ and $MD_{UF-IDE}=-\frac{1}{b}\text{log}\left(\frac{S_{2}}{S_{1}-N_{12}}\right)$, respectively, where $N_{12}=S_{1}-S_{2}\left(G=0\right)$ is obtained from the scout. One in vivo data set was denoised slice‐by‐slice using random matrix theory 43, implemented in MATLAB (window size = [8 8] voxels).

### INDI Sensitivity Simulations

This analysis aims to quantify the sensitivity of INDI with its equal temporal resolution dMRI counterpart. Non‐diffusion‐weighted INDI signals were computed through $S_{INDI}=\cos\left(\pi /4\right)*(1-e^{TR/T_{1}}$) for a broad range of TRs between 0.5 and 5 s and biologically relevant T_1_s between 0.5 and 2.5 s. The corresponding time‐matched dMRI signals with half the TR to maintain the same temporal resolution, were computed as $S_{dMRI}=\left(1-e^{\frac{TR}{2T_{1}}}\right)$. All simulations assume that magnetization has been prepared in a steady state by dummy scans.

## RESULTS

**Figure 2:**
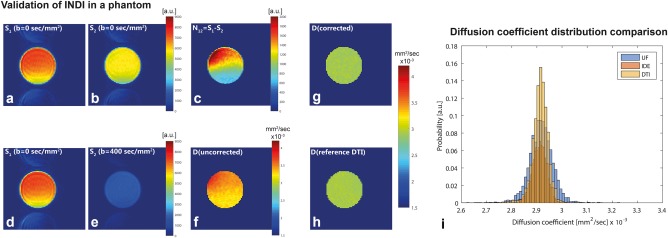
Experimental validation of INDI in a phantom. **a, b**: Raw data for the scout INDI image, representing S_1_ and S_2_ in the absence of diffusion weighting (ideally, S_1_ = S_2_). **c**: The difference image, N_12_, clearly shows that the echoes are not ideally matched. **d, e**: Raw data for INDI (specifically, UF‐IDE). The signal in (**e**) is significantly attenuated by diffusion. **f**: Mean diffusivity derived directly from the images in (**d**) and (**e**). The map is inhomogeneous, and the diffusion coefficient is larger than expected. **g**: Mean diffusivity calculated using a correction from the scout image, showing a homogenous image of the tube, as expected. **h**: Ground‐truth MD from DTI. Note that there is excellent agreement between the maps in (**g**) (single‐shot experiment) and (**h**) (12 different experiments are separated by a single TR for every image acquired). **i**: The UF‐IDE, IDE, and DTI histograms are clearly overlapping, suggesting excellent agreement among the methods and noisier data for UF‐IDE, as expected, at the fully relaxed condition.

The principles of INDI were first tested on a simple doped water phantom. Assuming T_1_ > > T_EPI/2_ + T_spoil_ (Fig. 1), S_1_ and S_2_(G = 0 mT/m) should ideally be identical for α=45° and β=90°; however, the two images are not exactly equal in practice (Figs. 2a and 2b). S_2_ signal intensity was typically somewhat weaker and spatially less homogeneous than S_1_, particularly in the phantom experiments, likely because of its very short T_1_/long T_2_ that can exacerbate effects of uncrushed coherence pathways, as well as potential B_1_ inhomogeneities, particularly for the π/4 pulse. Figure 2c shows the subtraction of the two signals, more clearly evidencing these differences (however, note that for example, in the in vivo experiments, the difference in these scout signals was much less pronounced, at approximately 5%).

Figures 2d and 2e show the raw data for a particular instantiation of INDI (i.e., the UF‐IDE experiment), showing the attenuation of S_2_ by diffusion weighting. Figure 2f shows the MD calculated directly from these raw images, without any correction applied. The uncorrected MD map suffers from two outstanding issues: (i) an artifactual spatial variation, unexpected for a homogeneous solution; and (ii) higher than expected MD values at this temperature. However, a simple subtraction of the scout image, N_12_, from S_1_ (as described in the Methods) completely remedies these discrepancies: The scout‐corrected MD coefficient map (Fig. 2g) is both homogeneous across the slice, and depicts the correct diffusion coefficient values as obtained from the gold standard DTI (Fig. 2h). Figure 2i and Supporting Table S1 further quantify the distribution of diffusion coefficients within the sample as obtained from the gold‐standard DTI, a conventional IDE, and the new UF‐IDE sequence. Clearly, all methods are in excellent agreement in this free diffusion scenario; however, as expected, a higher variance is observed for the UF‐IDE as a result of its inherently lower SNR in the fully relaxed regime.

**Figure 3:**
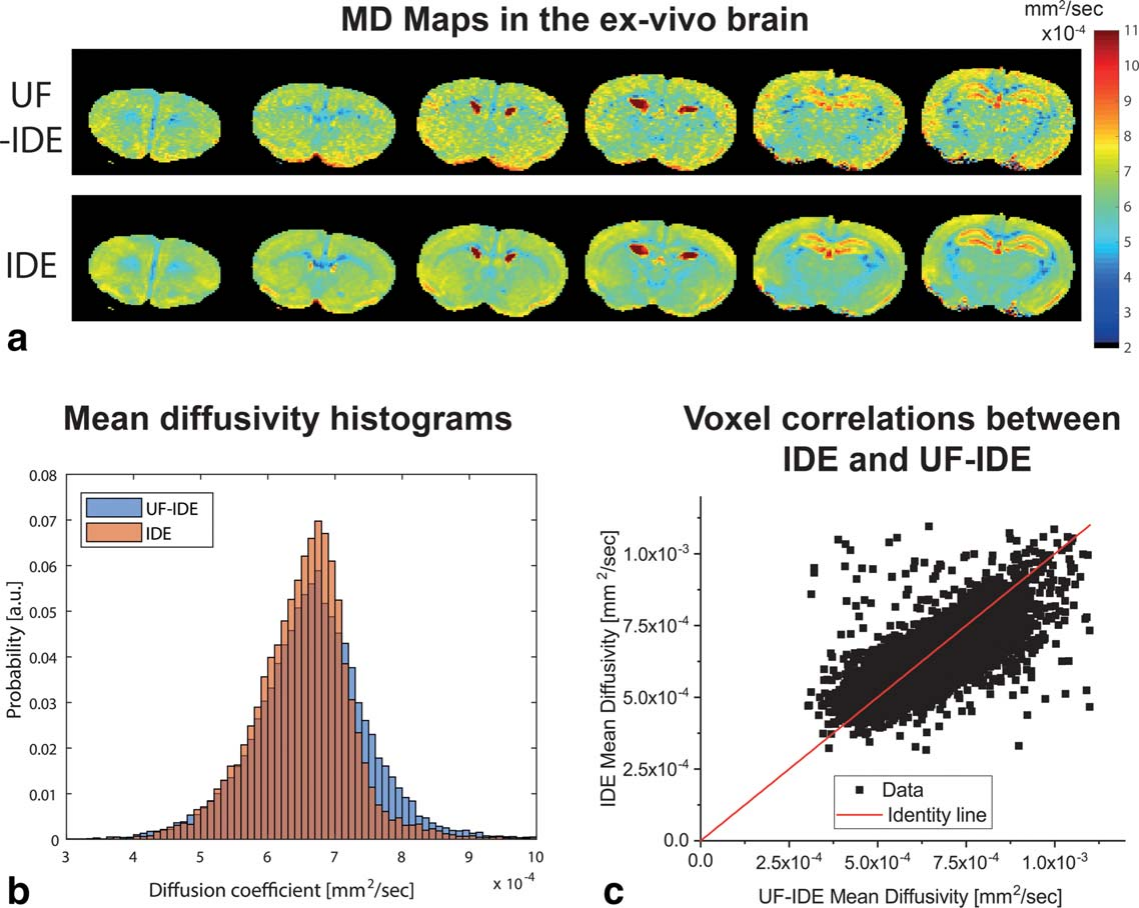
Validation of INDI in ex vivo brain. **a**: Mean diffusivity maps from UF‐IDE and IDE, showing comparable MD for UF‐IDE and IDE. **b**: Histogram analysis shows very similar distribution of MD for UF‐IDE and IDE. **c**: Correlation plot for UF‐IDE and IDE shows very good correspondence between the voxels acquired with different methods. All brain (but not surrounding) voxels were pooled together for both panels.

To test the applicability of INDI in a biological system, we performed similar experiments in ex vivo brains. Figure 3a shows MD maps derived from UF‐IDE (corrected with the N_12_ scout image) and from standard IDE in a representative brain. The UF‐IDE and conventional IDE experiments result in very similar MD maps, although the SNR is somewhat lower for UF‐IDE in this fully relaxed condition. Histograms from the entire brain are plotted in Figure 3b, whereas the median MD values arising from the different methods in the brain are tabulated in Supporting Table S1. The histograms are very similar for UF‐IDE and IDE, as are the median MD values. The true correspondence between UF‐IDE and its reference IDE was investigated by plotting the MD values in each voxel from the IDE experiments and their UF‐IDE counterparts (Fig. 3c). The plots are well‐correlated (Pearson's ρ = 0.71) with very high significance (uncorrected *P* < 1E−7).

**Figure 4:**
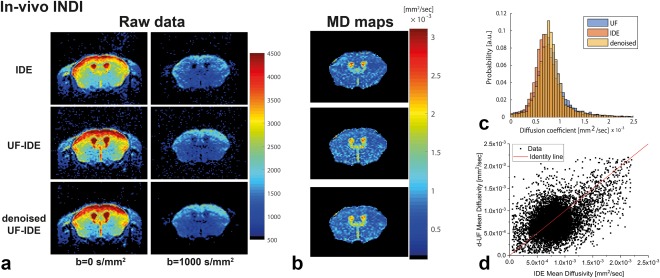
INDI in vivo. **a**: Raw data from a representative slice of the mouse brain acquired at 9.4 T, for conventional IDE (acquired with TR = 750 ms), UF‐IDE, and denoised IDE (d‐IDE), acquired with TR = 1500 ms, but having the same temporal resolution as the conventional IDE. Excellent image quality was observed. **b**: Corresponding MD maps. The single‐shot experiments are of good quality. **c**: Histogram distributions for the different methods for all brain (but not surrounding) voxels. The methods provide nearly identical distributions. **d**: Correlation analysis of IDE and d‐UF IDE reveals a good correlation among the methods.

To ensure that UF‐IDE can deliver robust images in vivo with high temporal resolution, experiments were performed on a mouse with a temporal resolution of 1.5 s (Fig. 4). The raw data (Fig. 4a) exemplify that the quality of UF‐IDE data are comparable with the corresponding temporally‐matched IDE, with approximately 20% higher SNR for the former. When denoised with random matrix theory 43, the image quality becomes even better, with SNR gains up to a factor of approximately 2 (Fig. 4a). The corresponding MD maps extracted from these experiments are shown in Figure 4b for a single slice and in Supporting Figure S1 for the rest of the slices acquired. The images are of high quality, considering the very high repetition rate. Histograms comparing the methods (Fig. 4c) overlap significantly, and the correlation between UF‐IDE and IDE (Fig. 4d) is highly significant (Pearson's ρ = 0.43, uncorrected *P* < 1E−7).

**Figure 5:**
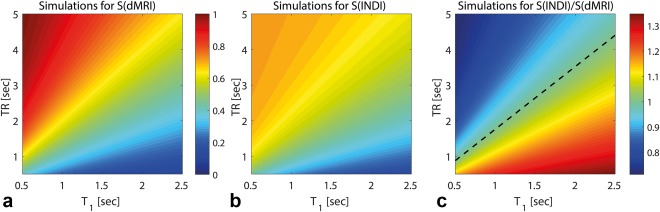
Signal‐to‐noise ratio analysis for INDI and conventional dMRI. Simulated signals for conventional dMRI (**a**) and INDI (**b**) over a wide range of practical TR values and T_1_ values are typical for biological tissues with field strengths between 1 and 16.4 T. Assuming constant noise with a standard deviation of one, the SNR profile of the two acquisitions is directly proportional to the signal maps. **c**: The INDI/dMRI signal ratios. The dashed line shows the point where dMRI and INDI have theoretically the same SNR, given that the two required images for each method are acquired with the same temporal resolution. The INDI method has a significant advantage when TR is less than approximately 1.76*T_1_.

To more directly compare the SNR properties of INDI and its conventional dMRI counterpart, Figures 5a and 5b illustrate non‐diffusion‐weighted signals (proportional to SNR up to a constant factor) for each method, for a broad range of TRs and biologically relevant T_1_s. Clearly, dMRI overperforms INDI for very long TRs; however, as TRs are decreased to approximately 1–2 s, the differences between the sequences' SNR becomes much less apparent. To analyze potential SNR enhancements by INDI, Figure 5c computes the ratio of S_INDI_/S_dMRI_. For short TRs invariably required in high temporal resolution applications, the dominance of hot colors shows a marked advantage of INDI over the equivalent dMRI experiment. Quantitatively, INDI will provide SNR gains as long as TR is less than approximately 1.76T_1_ (dashed line in Fig. 5c), although it should be noted that if INDI's scout images suffer signal loss, it will proportionally penalize SNR. Nevertheless, in our in vivo experiments, this was not an issue, and, in excellent agreement with the predictions of Figure 5c (TR of 1.5 s and T_1_ of ∼1.8 s), the nondenoised INDI acquisition indeed has an SNR gain of 1.20 to 1 when compared with the time‐matched IDE. Thus, INDI can be used to acquire the baseline and diffusion‐weighted images milliseconds apart, at least without suffering SNR loss, and potentially even with a modest SNR enhancement.

## DISCUSSION

Dynamic changes in tissue ADC are at the core of diffusion fMRI methods, aiming to map functional signals more intimately related with neural activity compared with their BOLD counterparts 30, 31, 44. Disentangling changes in diffusion‐driven metrics from changes in T_2_ on the TR timescale could potentially improve the characterization of dynamic microstructural changes. Here, we have described INDI, a single‐shot acquisition scheme with an inherent robustness against T_2_ changes occurring on the TR timescale. By harnessing a partial initial nutation of the magnetization to encode the baseline image, it is possible to acquire the diffusion‐weighted image only milliseconds later using the unperturbed magnetization reservoir. The TR is then fruitfully used to recover magnetization and reduce T_1_ weighting. The INDI features were exemplified in a water phantom, in which a single, time‐independent diffusion coefficient exists, and was accurately extracted from UF‐IDE experiments. Both ex vivo and in vivo brain experiments evidenced very good correspondence between IDE and UF‐IDE data.

It is instructive to consider INDI's SNR regimes. For initial conditions satisfying fully relaxed magnetization, the ideal INDI as prescribed here will incur a penalty of $\cos\left(\frac{\pi }{4}\right)=\frac{\sqrt{2}}{2}M_{0}$, whereas the corresponding dMRI will of course make use of the entire *M*_*0*_. However, rapid acquisition schemes invariably entail non–fully relaxed conditions, in which INDI's magnetization (assumed to be set to an initial steady state by dummy scans) will have decayed by a factor of $\frac{\sqrt{2}}{2}*\left(1-e^{-\frac{TR}{T_{1}}}\right)$, whereas the temporally equivalent dMRI would decay by $1-e^{-\frac{TR}{2T_{1}}}$; the factor of 0.5 in the latter exponential accounts for acquiring two dMRI images with identical temporal resolution as INDI. Theoretically, it can be shown that for $TR<∼1.76T_{1}$, $S_{INDI}>S_{dMRI}$, and as shown in Figure 5c, for most biologically relevant conditions, INDI could even entail moderate sensitivity enhancements.

The INDI scout images also deserve some discussion for their temporal stability. The scout is used to normalize each pair of INDI images along a time series, thereby implicitly assuming that motion effects are negligible. However, in some applications, such as heart imaging, this assumption may be severely violated. In these cases, several scout images could be acquired in cine mode (i.e., with their cycle phase‐locked to some external trigger and every INDI experiment measured along the cycle corrected with its phase‐locked counterparts scout). Another alternative is to entirely forego the scout. Although the absolute value of MD may be biased, its time course may still be of significant value, as the bias should be constant, assuming that T_2_ does not vary on the millisecond timescale. Finally, although we presented scout images with α=45° and β=90°, the difference between the baseline images can be made even smaller if the specific values for the first and second nutation pulses are tweaked (data not shown). For example, although we showed worst‐case scenarios for the tube of water, the brain's scout images differed by approximately 5%, which could be mitigated even further with tweaking of the nutation angles (data not shown). If a good balance between the scout's S_1_ and S_2_ is achieved, then the scout images are not required, and the INDI experiment can proceed without the normalization step.

Here, we focused on a specific implementation of INDI (i.e., the UF‐IDE sequence), and demonstrated its feasibility and utility for assessing MD. However, INDI can be used with any gradient waveform such as double‐diffusion encoding (14, 15, 45] or nonuniform oscillating‐gradient spin echo 46.

In conclusion, the INDI pulse sequence was presented and revealed its capability of mapping accurate diffusion coefficients with good sensitivity and excellent temporal resolution. The feasibility of INDI in preclinical settings was demonstrated, and its immunity toward rapid changes in T_2_ are promising for future dfMRI experiments and other applications calling for rapid mapping of microstructural dynamics.

## Supporting information


**Fig. S1.** Initial nutation diffusion imaging maps in vivo for all slices. Top to bottom rows represent IDE, UF‐IDE, and the denoised UF‐IDE MD maps, respectively.
**Table S1.** Median and Interquartile Ranges for the MD Extracted From the Different Methods.
