# Supplementary material for: Incomplete initial nutation diffusion imaging: An ultrafast, single‐scan approach for diffusion mapping
Source: Magn Reson Med. 2017 Sep 3;79(4):2198–204. doi: 10.1002/mrm.26894 (PMC5836954; doi:10.1002/mrm.26894)
Supplement: Supplementary file 1 — Fig. S1. Initial nutation diffusion imaging maps in vivo for all slices. Top to bottom rows represent IDE, UF‐IDE, and the denoised UF‐IDE MD maps, respectively. Table S1. Median and Interquartile Ranges for the MD Extracted From the Different Methods. [file MRM-79-2198-s001.docx]

**Supporting Material:**

**Incomplete Nutation Diffusion Imaging: an ultrafast, single-scan approach for diffusion mapping**

Andrada Ianuş^1,2^ and Noam Shemesh^1^*

^1^Champalimaud Neuroscience Programme, Champalimaud Centre for the Unknown, Lisbon, Portugal

^2^Centre for Medical Image Computing, Dept. of Computer Science, University College London, London, UK.

*Corresponding author

Noam Shemesh

Champalimaud Neuroscience Programme, Champalimaud Centre for the Unknown

Av. Brasilia 1400-038

Lisbon, Portugal

E-mail: noam.shemesh@neuro.fchampalimaud.org

Phone number: +351 210 480 000 ext. #4467

**Contents:**

1. Supporting Theory

2. Supporting Table

3. Supporting Figure

4. Supporting References

**Theory**

INDI framework is presented in Figure 1. We assume for simplicity only on-resonant magnetization, ideally homogeneous magnetic field, and instantaneous perfect RF pulses. Given equilibrium magnetization $\left\{ M_{x},M_{y},M_{z} \right\}=\{0,0,M_{0}\}$, an arbitrary pulse of flip angle $\alpha_{x}<\frac{\pi}{2}$ (where *x* denotes the pulse phase) will rotate the magnetization such that immediately after the pulse,

$\left\{ M_{x},M_{y},M_{z} \right\}_{\alpha_{x}}=\{0,-M_{0}\sin\left( \alpha\right), M_{0}cos(\alpha)\}$.

In the rotating frame of reference, the in-plane magnetization will then simply decay with T_2_ and the longitudinal magnetization will start relaxing slowly with T_1_. The refocusing pulse along the y-axis,$\pi_{y}$, will not affect the in-plane magnetization, but will invert the longitudinal component, and thus the magnetization immediately following the refocusing pulse will be

$\left\{ M_{x},M_{y},M_{z} \right\}_{\pi_{y}}=\{0,-M_{0}\sin\left( \alpha\right)e^{-\frac{TE}{2T_{2}}},{-M}_{0}[\left( \cos\left( \alpha\right)-1 \right)e^{-\frac{TE}{2T_{1}}}+1]\}$.

At the first echo the magnetization components will evolve to

$\left\{ M_{x},M_{y},M_{z} \right\}_{TE1}=\{0,-M_{0}\sin\left( \alpha\right)e^{-\frac{TE}{T_{2}}}, M_{0}[1-2e^{-\frac{TE}{{2T}_{1}}}+(1-\cos\left( \alpha\right))e^{-\frac{TE}{T_{1}}}]\}$.

The measured signal around the echo is assumed to be acquired with ultrafast acquisitions, e.g., with EPI [[1](#_ENREF_1)] or spiral [[2](#_ENREF_2)]. Since only a fraction of the magnetization (proportional to the flip angle α) is manipulated during the first acquisition, a fresh reservoir of longitudinal magnetization will be available immediately for another experiment. This is the core idea of INDI in general, and the specific pulse sequence presented here in particular: if two images differing only by diffusion weighting are required to obtain the MD map, then the second signal can be obtained from this fresh magnetization. Hence, assuming that very little longitudinal relaxation will practically occur due to TE << T_1_ and following crushing any residual magnetization (which in our case was done with a strong gradient (that, in our case, was thereafter rewound to avoid phase twisting, though this was later determined as non-critical), the sequence continues without delay with a new nutation pulse, $\beta$. We choose $\beta_{-x}=\frac{\pi}{2}$ to convert all the reservoir $M_{z}$ magnetization to detectable magnetization with the same phase as the previous echo. An identical spin-echo sequence is then repeated, with identical timing as before, only now the diffusion gradients are played out, in our specific case – we chose an IDE waveform (this particular INDI variant will be here termed ultrafast-IDE (UF-IDE). Since the echo times are identical and it can be assumed that very little longitudinal relaxation occurs between the two acquisitions, the first signal provides the baseline image, while the second comprises an IDE-weighted image. Mean diffusivity follows trivially from $MD=-log(\frac{S_{2}}{S_{1}})/b$, where $S_{i}$ denote each image obtained in UF-IDE, and *b* is the total b-value of the IDE waveform. The INDI method is especially tailored for scenarios where T_2_ may vary in the time scale of TR, as both S_1_ and S_2_ are acquired only a few milliseconds apart: this means that if T_2_-weighting varies between the TRs (but assumed to be stable over a few milliseconds), then the MD extracted will be normalized with respect to the T_2_ variations.

Ideally, given a choice of flip angles $\alpha=45^{\circ}$ and $\beta=90^{\circ}$, the two images from an INDI acquisition without diffusion weighting (G = 0 mT/m) should be almost identical, assuming T_1_ >> TE + T_spoil_, where TE is the echo time and T_spoil_ is the time interval between the first echo and the second excitation pulse (Figure 1). However, the two images may not exactly the same in practice. In order to account for any differences between S_1_ and S_2_(G = 0 mT/m), a “scout” sequence can be acquired once prior to running the INDI experiments, with otherwise identical experimental parameters as the actual UF-IDE, except with no diffusion gradients at all. For these cases, the normalizing image resulting from the scout, $N_{12}={S_{1}-S}_{2}(G=0)$, can be used to correct the subsequent times series through correcting the mean diffusivity with $MD_{UF-IDE}=-\frac{1}{b}log(\frac{S_{2}}{S_{1}-N_{12}})$. Note that this subtraction implicitly assumes linearity and that variations in T_2_ do not occur on the timescale TE + T_spoil_.

Supporting Table S1. Median and interquartile ranges for the mean diffusivity extracted from the different methods^1^

| **Sample/Method** | **UF-IDE^a^** | **IDE^b^** | **DTI^c^** |
| --- | --- | --- | --- |
| Water phantom | 2.92±0.06 | 2.91±0.04 | 2.92±0.04 |
| Ex-vivo Brain | 0.67±0.10 | 0.66±0.08 | - |

^a^Single shot

^b^Required two images separated by a single TR

^c^Measured with twelve images, each separated by TR

**
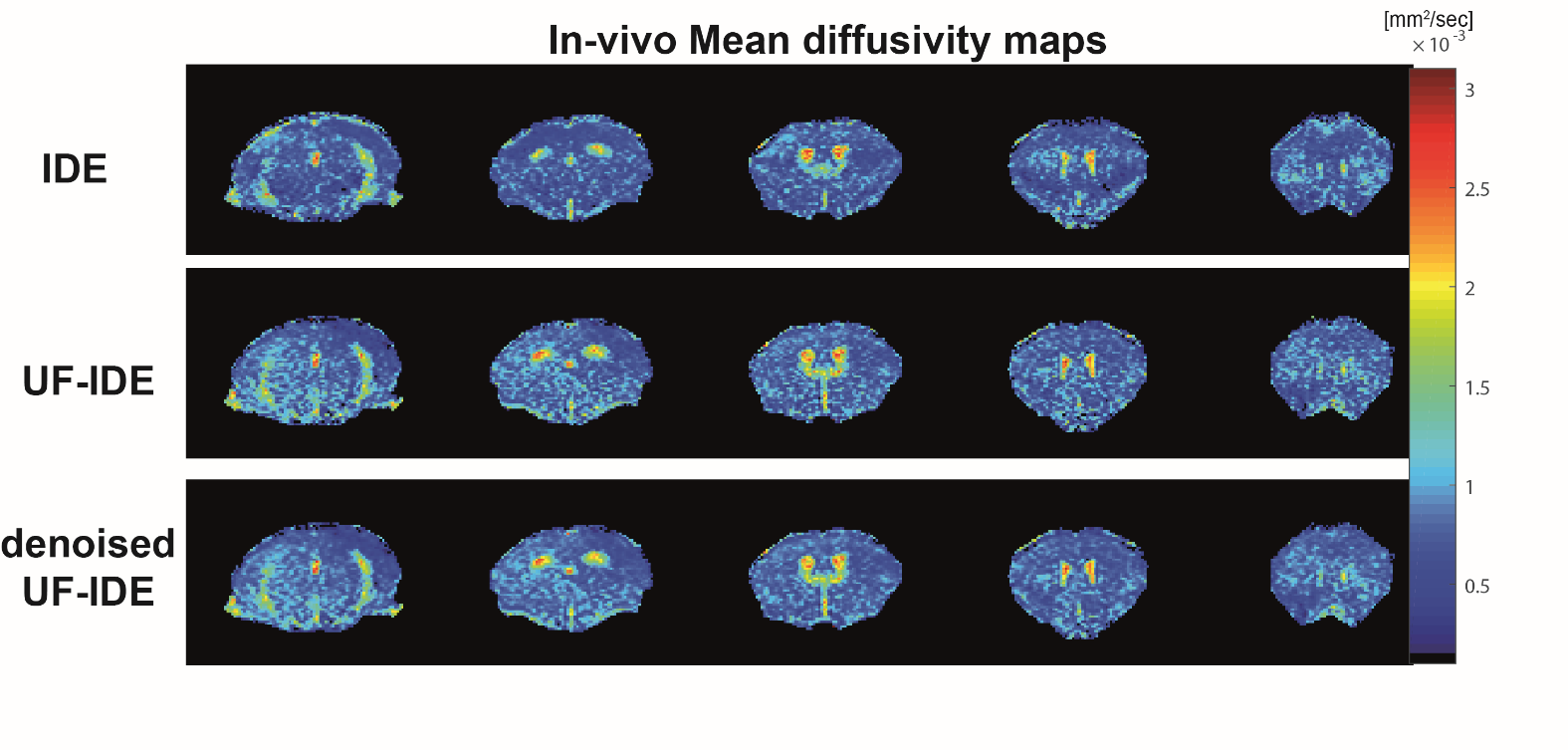
**

**Supporting Figure S1.** **INDI maps in-vivo for all slices.** Top to bottom rows represent IDE, UF-IDE, and the denoised UD-IDE mean diffusivity maps.

**Supplementary References**

1. Stehling, M.K., R. Turner, and M. Mansfield, *Echo-Planar Imaging: Magnetic Resonance Imaging in a Fraction of a Second.* Science, 1991. **254**: p. 43-50.

2. Mayer, C., et al., *Fast Spiral Coronary Artery Imaging.* Magn Reson Med, 1992. **28**: p. 202-213.
